# Association between prostate cancer and susceptibility, hospitalization, and severity of COVID-19: Based on a Mendelian randomization study

**DOI:** 10.1097/MD.0000000000039430

**Published:** 2024-09-06

**Authors:** Jingwen Liu, Lijun Wan, Jianyong Zhu, Renbing Pan

**Affiliations:** a Longyou People’s Hospital Affiliated with Sir Run Run Shaw Hospital, Zhejiang University School of Medicine, Quzhou, Zhejiang, China; b Department of Urology, The Quzhou Affiliated Hospital of Wenzhou Medical University, Quzhou People’s Hospital, Quzhou, Zhejiang, China.

**Keywords:** COVID-19, Mendelian randomization, prostate cancer, single nucleotide polymorphisms, TMPRSS2

## Abstract

Several observational studies indicated a close association between prostate cancer and COVID-19. Nevertheless, whether there was a causal effect between them remained obscure. In this study, we aimed to detect the potential association between genetically determined prostate cancer and the risk of COVID-19. A bidirectional Mendelian randomization (MR) study was conducted to investigate the causal links between prostate cancer and COVID-19. Inverse variance weighted (IVW), MR-Egger, weighted median, weighted mode, and simple mode were used to estimate the causality. P_IVW_ < 0.05 was considered statistically significant. The top single nucleotide polymorphisms associated with prostate cancer cases (n = 79,148) and COVID-19 cases (n = 54,071) were extracted from the summary genome-wide association study data obtained from a publicly available database. Cochran Q test was utilized to calculate the degree of heterogeneity. Additionally, we validated our findings in another replication cohort. In the forward MR study, the IVW method suggested no evidence for the causal effect of prostate cancer on COVID-19 susceptibility (OR = 1.00, 95%CI: 0.98–1.02, *P* = .978), COVID-19 hospitalization (OR = 1.05, 95%CI: 0.99–1.09, *P* = .054), and COVID-19 severity (OR = 1.03, 95%CI: 0.95–1.11, *P* = .453). Reverse MR analysis also showed no causal effect of COVID-19 diverse phenotypes on prostate cancer. Furthermore, the result of the East Asian cohort study was consistent with the European cohort. Sensitivity analysis showed no evidence of pleiotropy and heterogeneity. We did not discover genetic evidence to substantiate causal links between prostate cancer and COVID-19. Large-scale randomized controlled trials were required to enhance a more profound comprehension of this relationship in the future.

## 1. Introduction

The coronavirus disease 2019 (COVID-19), resulting from infection with severe acute respiratory syndrome coronavirus 2 (SARS-CoV-2) infection, has instigated a global state of alarm and led to enormous economic and health burdens.^[1]^ The ongoing COVID-19 pandemic has affected millions globally and presented a grievous public health threat.^[2]^ The people who died from COVID-19 were elderly individuals, those with chronic obstructive pulmonary disease, diabetes mellitus, and cancer alike.^[3]^ Cancer and COVID-19 manifested clinical and molecular similarities in some signaling pathways, such as type I interferon, cytokine, immune checkpoint signaling, and androgen receptor.^[4]^

Indeed, the correlation between COVID-19 and cancers has garnered considerable attention. Due to the immunosuppressive effect of cancer-related therapy and the disease itself, individuals with cancer were at a heightened risk of developing life-threatening complications from COVID-19.^[5,6]^ Current studies have shown that a close correlation between prostate cancer and COVID-19.^[7]^ Dimple Chakravarty et al^[8]^ conducted observational studies and discovered that the susceptibility to COVID-19 was found to be higher in individuals diagnosed with prostate cancer. The rates of hospitalization and mortality were higher in COVID-19 patients with prostate cancer than those with non-prostate genitourinary malignancies. Additionally, another retrospective study reported that COVID-19 severity was correlated with elevated levels of pro-inflammatory regulators and cytokine storm.^[9]^ The inflammatory cytokines produced by COVID-19 infection and prostate cancer were different from each other. The pro-inflammatory cytokines induced by COVID-19 can exacerbate prostate cancer.^[10]^ Nevertheless, the link between COVID-19 and prostate cancer in observational studies might be subject to bias due to confounders and reverse causality.^[11]^ Thus, it was still unclear whether prostate cancer was the cause or result of COVID-19.

Mendelian randomization (MR) approach, based on genome-wide association studies (GWAS), was a robust method for constructing instrumental variables (IVs) and inferring causality between 2 traits in which a causal link was difficult to establish utilizing the retrospective and observational study.^[12]^ Furthermore, MR played a crucial role in disentangling causal relationships from associations influenced by confounding factors and reverse causal bias.^[13]^ The MR study provided evidence of causality that bridged the gap between conventional epidemiological studies and randomized controlled trials.^[14]^ In this study, we conducted a bidirectional MR analysis using summary statistics from GWAS in the European population to detect the underlying causality between prostate cancer and COVID-19 and validated the findings in individuals of East Asian ancestry. Understanding the bidirectional correlation between prostate cancer and COVID-19 is of great public health importance in complications management and disease prevention.

## 2. Materials and methods

### 2.1. Study design and assumption

**Figure 1.**
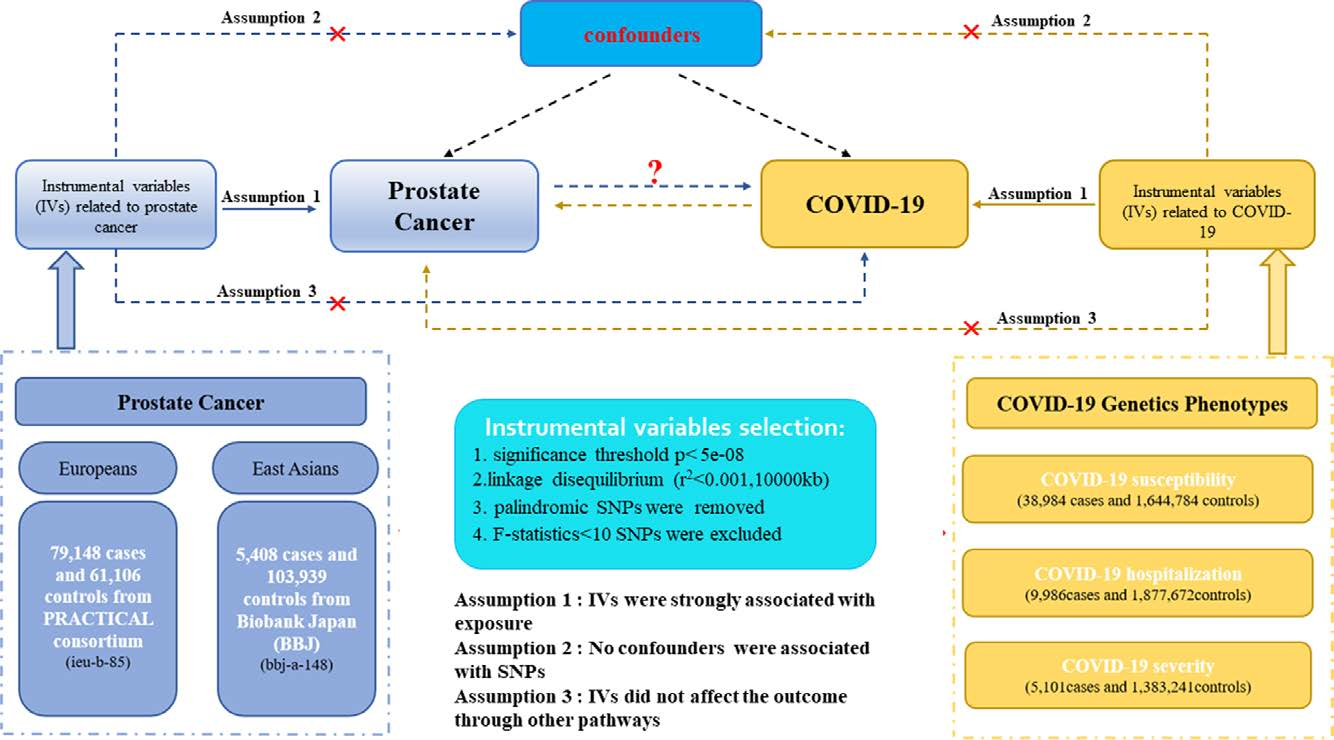
Description of the datasets, assumptions, and study design in this bidirectional MR study. The blue represented the forward MR analysis, with prostate cancer as exposure and COVID-19 as the outcome. The yellow represented the reverse MR analysis, with COVID-19 as exposure and prostate cancer as the outcome. COVID-19 = coronavirus disease 2019, MR = Mendelian randomization.

Figure 1 demonstrates a clear workflow of this bidirectional MR design between prostate cancer and COVID-19 diverse phenotypes. Our study employed the publicly available data from GWAS on prostate cancer and COVID-19 phenotypes, with detailed characteristics and information listed in Table S1, Supplemental Digital Content, http://links.lww.com/MD/N438. The forward MR analyses considered prostate cancer as the exposure and COVID-19 as the outcome, while the reverse MR analyses COVID-19 as exposure and prostate cancer as the outcome. Relevant single nucleotide polymorphisms (SNPs) were extracted from the GWAS summary data through the implementation of quality control procedures. To augment the reliability of the results, this MR study endeavored to satisfy the following 3 assumptions. (1) IVs are strongly associated with the exposure. (2) No confounders are associated with the genetic proxy of exposure (SNP). (3) IVs influence the outcome only through exposure, and there are no alternative causal pathways for the genetic IVs to impact the outcome. All of the information utilized in this study was derived exclusively from publicly accessible GWAS summary statistics that had been publicly published.

### 2.2. Data sources

#### 2.2.1. Prostate cancer

The summary data set for prostate cancer was derived from the largest GWAS meta-analysis, which included 79,148 cases and 61,106 controls of European descent from the PRACTICAL consortium.^[15]^ To each GWAS, the analysis was adjusted for both specific principal components and relevant covariates, while the overall meta-analysis accounted for principal components. This meta-analysis datasets contain 20,346,368 SNPs. Prostate cancer individuals were diagnosed by experienced surgeons at each cooperating institution. The diagnosed data was collected through medical records.

#### 2.2.2. COVID-19 phenotypes

The GWAS statistics with COVID-19 were obtained from the COVID-19 Host Genetics Initiative platform,^[16]^ which is an international collaboration aiming to facilitate COVID-19 genetics research. The COVID-19 data we selected exclusively consisted of participants of European ancestry and had the largest sample size. Eventually, 3 COVID-19 phenotypes were selected from the GWAS datasets, including susceptibility (cases: 38,984; controls: 1,644,784), hospitalization (cases: 9986; controls: 1,877,672), and severity (cases:5101; controls: 1,383,241). The profile of these COVID-19 phenotypes data is presented in Table S1, Supplemental Digital Content, http://links.lww.com/MD/N438. In the dataset, the COVID-19 susceptibility cases were defined as individuals who had laboratory confirmation of SARS-CoV-2 infection based on nucleic acid test. The COVID-19 hospitalization was defined as individuals infected with SARS-CoV-2 who required admission to a medical facility due to symptoms associated with COVID-19. The COVID-19 severity was defined as the hospitalization of participants with COVID-19 exhibiting severe respiratory symptoms and needing respiratory support, such as intubation, continuous positive airway pressure, or bilevel positive airway pressure.^[17]^ All of these participants belonged to patients with COVID-19 infections and were from the European population in 2020. The infectious strain of this period was basically the Delta strain and none of the participants were vaccinated. The patients were identified based on laboratory-confirmed infection, electronic health records, clinical diagnosis by a healthcare professional, or self-reporting.

### 2.3. Selection of instrumental variables

Firstly, independent SNPs with genome-wide significance threshold (*P* < 5e‐08, which indicates a strong association between IVs and exposures) with exposure were used as instruments. Secondly, the potential impact of linkage disequilibrium between SNPs was mitigated by implementing an linkage disequilibrium threshold for the selected SNPs (*r*^2^ < 0.001, 10,000 kb), ensuring the independence of IVs for each exposure. Thirdly, we figured out the F-statistics to assess the IVs’ strength, and excluded weak IVs with F < 10.^[18]^ Furthermore, those IVs associated with the outcome with *P* < 5e‐08 were excluded, and intermediate allele frequency palindromic SNPs were also removed. Additionally, to satisfy the second assumption of MR, SNPs associated with confounding factors, including blood pressure, body mass index, and smoking, were excluded by applying the PhenoScannerV2 online platform.

### 2.4. Statistical analysis

To explore the links between prostate cancer and COVID-19, 5 approaches, encompassing MR-Egger, weighted median, inverse variance weighted (IVW), simple mode, and weighted mode were used to investigate the causality between exposure and outcome.^[19]^ As a meta-analysis approach, the random-effects IVW method was used as the dominant means to assess the underlying bidirectional causal links between COVID-19 and prostate cancer. The IVW method is predicated on the assumption that 3 fundamental hypotheses hold.^[20]^ The significance level was adjusted using the Bonferroni correction to account for multiple comparisons (*P* = .05/3 = .017). This adjustment allowed us to identify a significant causal relationship among the 3 different COVID-19 phenotypes. In the forward MR analyses, the exposure variable was assigned to prostate cancer, while the outcome variable was assigned to COVID-19 phenotypes, and in the reverse MR analyses vice versa. Additionally, other analysis methods were also applied as complementary approaches.

### 2.5. Sensitivity analyses

The sensitivity analyses were conducted including MR pleiotropy residual sum and outlier (MR-PRESSO), MR-Egger regression, Cochran Q test, and leave-one-out analysis. Examination of pleiotropy was employed by MR-Egger regression analyses. The significant causal association should also exhibit a nonsignificant MR-Egger intercept (*P* > .05), indicating the absence of horizontal pleiotropy.^[21]^ MR-PRESSO test was employed to explore any outliers that could result in pleiotropic biases and to control for the effects of pleiotropy by removing these SNPs. Cochran Q statistic was computed to quantify the heterogeneities identified by the IVW and MR-Egger regression approach, with a *P*-value >.05, indicating no presence of heterogeneity among the IVs. In addition, we further examined whether some SNPs created bias to affect the overall causal estimate independently and assessed the stability of effect sizes via leave-one-out analysis.

All the MR analysis and sensitivity analysis were performed with R (version 4.2.3). R packages “Two Sample MR” and “MR-PRESSO” packages were utilized.

### 2.6. Secondary validation study

We further perform a secondary validation study for bidirectional MR analysis based on another independent prostate cancer GWAS data set from East Asian descent. The GWAS data of the validation cohort were derived from Biobank Japan, including 5408 prostate cancer cases and 103,939 healthy controls.^[22]^ The detailed information and traits of GWAS data are displayed in Figure 1 and listed in Table S1, Supplemental Digital Content, http://links.lww.com/MD/N438.

### 2.7. Ethical statement

All data in this study were obtained from open publicly attainable database. We did not obtain these data from patients directly or intervene in these patients. Thus, ethical approval and written informed consent were not required for this study.

## 3. Results

### 3.1. Selection of instrumental variables

After proxy SNP exploration, palindromic SNP removal, and the PhenoScannerV2 database screening, we extracted eligible SNPs as IVs to satisfy 3 core assumptions. In the forward MR, the number of SNPs was 120 for European prostate cancer and 22 for East Asian prostate cancer. The F-statistic for every SNP was >10. The explicit information of SNPs for European cohort and East Asian cohort were respectively demonstrated in Tables S2, Supplemental Digital Content, http://links.lww.com/MD/N440 and S3, Supplemental Digital Content, http://links.lww.com/MD/N441. In addition, Venn diagram exhibited the shared SNPs between the European cohort and East Asian cohort (Table S4, Supplemental Digital Content, http://links.lww.com/MD/N442).

### 3.2. Causal effect of prostate cancer on COVID-19 via forward MR

**Figure 2.**
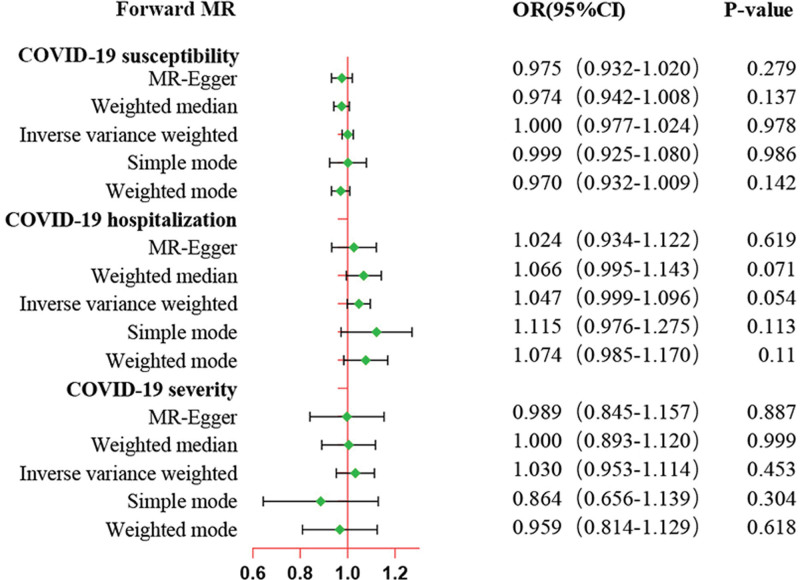
MR estimates of genetically predicted prostate cancer on the susceptibility, hospitalization, and severity of COVID-19 in Europeans. The inverse variance weighted (IVW) method is considered the dominant method. COVID-19 = coronavirus disease 2019, MR = Mendelian randomization

Forward MR analysis results showed no significant evidence for a causal effect of prostate cancer on COVID-19 diverse phenotypes. As shown in Figure 2 and Table S5, Supplemental Digital Content, http://links.lww.com/MD/N443, the IVW method suggested no evidence for the causal effect of prostate cancer on COVID-19 susceptibility (OR = 1.00, 95%CI: 0.98–1.02, *P* = .978), COVID-19 hospitalization (OR = 1.05, 95%CI: 0.99–1.09, *P* = .054), and COVID-19 severity (OR = 1.03, 95%CI: 0.95–1.11, *P* = .453). In addition, no significant association was identified in other methods. Horizontal pleiotropy was not observed in the intercept of MR-Egger regression (Table 1, *P* > .05). Furthermore, Cochran Q test indicated significant heterogeneity (*P* < .05) for IVW and MR-Egger approach when explored the MR estimate of prostate cancer on COVID-19 severity, so we performed the IVW method with random-effect model (Table 1). The forest plots, scatter plots, leave-one-out results and funnel plots of genetically predicted prostate cancer on COVID-19 were presented in Figures S1, Supplemental Digital Content, http://links.lww.com/MD/N424, S2, Supplemental Digital Content, http://links.lww.com/MD/N426, S3, Supplemental Digital Content, http://links.lww.com/MD/N428, S4, Supplemental Digital Content, http://links.lww.com/MD/N429. The MR-PRESSO global test manifested no horizontal pleiotropy between the IVs and outcomes, and no outlier was identified (Table 1).

**Table 1:** Heterogeneity and horizontal pleiotropy analyses results.

Exposure	Outcome	Horizontal pleiotropy				Heterogeneity			
		MR-PRESSO global outlier test		MR-Egger regression		MR-Egger		IVW	
		*P*-value	Outlier	Intercept	*P*-value	Q-statistic	*P*-value	Q-statistic	*P*-value
COVID-19 susceptibility	Prostate cancer	.621	No	0.0028	.920	21.60	.001	21.65	.001
COVID-19 hospitalization	Prostate cancer	.725	No	‐0.00024	.978	1.172	.759	1.173	.882
COVID-19 severity	Prostate cancer	.733	No	‐0.0049	.809	21.44	.002	21.67	.003
Prostate cancer	COVID-19 susceptibility	.625	No	0.0029	.201	128.87	.161	130.74	.149
Prostate cancer	COVID-19 hospitalization	.264	No	0.0025	.581	124.25	.221	124.58	.234
Prostate cancer	COVID-19 severity	.412	No	0.0046	.553	150.59	.012	151.38	.013

### 3.3. Causal effect of COVID-19 on prostate cancer via reverse MR

**Figure 3.**
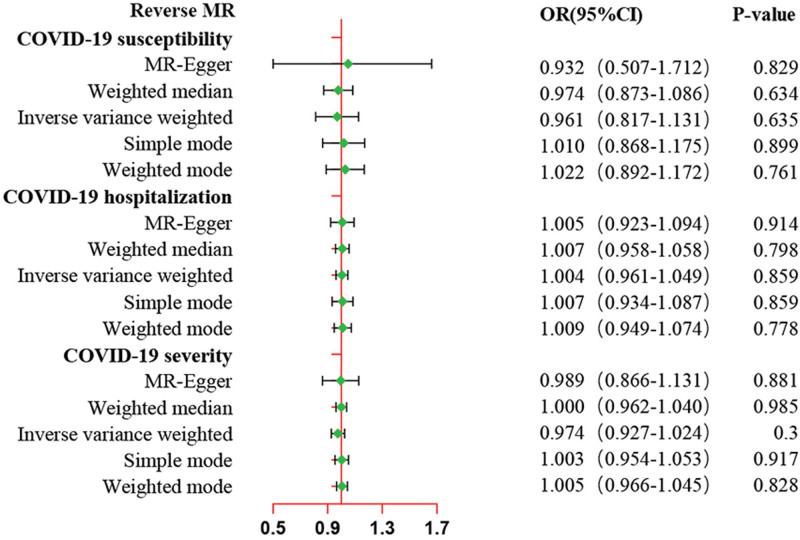
MR estimates of genetically predicted susceptibility, hospitalization, and severity of COVID-19 on prostate cancer in Europeans. The inverse variance weighted (IVW) method is considered the dominant method. COVID-19 = coronavirus disease 2019, MR = Mendelian randomization

Reverse MR analysis was performed to investigate the causal effects of COVID-19 various phenotypes on prostate cancer and yielded similar findings. As shown in Figure 3 and Table S6, Supplemental Digital Content, http://links.lww.com/MD/N444, the IVW method showed no significant evidence for the causal effect of COVID-19 susceptibility on prostate cancer (OR = 0.96, 95%CI: 0.82–1.13, *P* = .635), nor did COVID-19 hospitalization (OR = 1.00, 95%CI: 0.96–1.05, *P* = .859) and COVID-19 severity (OR = 0.97, 95%CI: 0.93–1.02, *P* = .300). Moreover, the null results were confirmed in the evaluation of other approaches. An MR-Egger intercept was performed, and the test suggested no horizontal pleiotropy (Table 1, *P* > .05). Additionally, Cochran Q test exhibited heterogeneity among the selected SNPs when investigated the causal effects of COVID-19 susceptibility and severity on prostate cancer (*P* < .01), as shown in Table 1. The MR-PRESSO global test demonstrated no horizontal pleiotropy and no outlier was explored (Table 1). The forest plots, scatter plots, leave-one-out results, and funnel plots of genetically predicted COVID-19 on prostate cancer were shown in Figures S1, Supplemental Digital Content, http://links.lww.com/MD/N424, S2, Supplemental Digital Content http://links.lww.com/MD/N426, S3, Supplemental Digital Content, http://links.lww.com/MD/N428, and S4, Supplemental Digital Content http://links.lww.com/MD/N429.

### 3.4. East Asian population validation results

**Figure 4.**
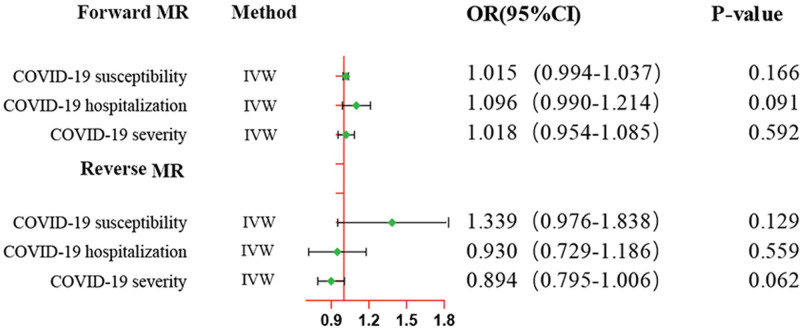
Genetic causal associations between prostate cancer and COVID-19 phenotypes in East Asians. The inverse variance weighted (IVW) method is considered the dominant method. COVID-19 = coronavirus disease 2019.

In the East Asian participants’ validation cohort, we also conducted a bidirectional MR analysis. For the forward MR analysis, the results of MR analysis manifested that genetically predicted prostate cancer was not causally associated with COVID-19 susceptibility (OR = 1.02, 95%CI: 0.99–1.04, *P* = .166), COVID-19 hospitalization (OR = 1.09, 95%CI: 0.99–1.21, *P* = .091), and COVID-19 severity (OR = 1.02, 95%CI: 0.95–1.09, *P* = .592). Additionally, for the reverse MR analysis, no causal effects of COVID-19 susceptibility (OR = 1.34, 95%CI: 0.98–1.84, *P* = .129), COVID-19 hospitalization (OR = 0.93, 95%CI: 0.73–1.19, *P* = .559), and COVID-19 severity (OR = 0.89, 95%CI: 0.80–1.01, *P* = .062) on prostate cancer were found. Furthermore, MR-Egger and IVW Cochran Q tests indicated no significant heterogeneity of IVs (all *P*-values > .05). The sensitivity analysis did not reveal any pleiotropy, and the results of leave-one-out sensitivity analysis were not influenced by any individual SNPs (Fig. S7, Supplemental Digital Content, http://links.lww.com/MD/N433). The results of the MR analysis between East Asian prostate cancer and COVID-19 were displayed in Figure 4 and Tables S7, Supplemental Digital Content, http://links.lww.com/MD/N446 and S8, Supplemental Digital Content, http://links.lww.com/MD/N448. Moreover, the forest plots, scatter plots, and funnel plots of the causal links between genetically determined prostate cancer and COVID-19 were demonstrated in Figures S5, Supplemental Digital Content, http://links.lww.com/MD/N430, S6, Supplemental Digital Content, http://links.lww.com/MD/N431, and S8, Supplemental Digital Content, http://links.lww.com/MD/N436.

## 4. Discussion

To our knowledge, this is the first study to explore the causal relationship between prostate cancer and COVID-19 with summary GWAS data. Our two-sample MR analysis failed to find any evidence supporting the causal association of prostate cancer with COVID-19 in populations of European ancestry. Similarly, the reverse MR analysis suggested no evidence that genetically predicted COVID-19 was causally linked to prostate cancer. Additionally, replicated analyses in the East Asian cohort yielded consistent findings that there was no causal association between prostate cancer and COVID-19.

In 2019, the World Health Organization proclaimed COVID-19 to be a global pandemic.^[23]^ Patients with COVID-19 infection may occasionally also have prostate cancer. The immunosuppressed condition of prostate cancer patients has been demonstrated in several observational studies to render them more susceptible to COVID-19 compared to healthy individuals, as previously mentioned.^[24]^ Nevertheless, due to the special androgen deprivation therapy (ADT) of prostate cancer, previous observational studies demonstrated that the COVID-19 patients with ADT had better clinical outcomes.^[25]^ Another observational study suggested that the clinical outcomes of individuals with prostate cancer who were infected with COVID-19 were poorer compared to those of patients with non-prostate genitourinary malignancies.^[26]^ In addition, previous studies also indicated that the progression of prostate cancer can be enhanced by several pro-inflammatory cytokines induced by COVID-19.^[10]^ These studies have focused on the potential association between prostate cancer and COVID-19, but no one has revealed the confirmative causal link. Herein, we assessed the causality between COVID-19 and prostate cancer by conducting two-sample MR analyses. Unexpectedly, in both the European cohort and the East Asian cohort, our study demonstrated no evidence supporting a causal relationship between prostate cancer and an increased risk of COVID-19. In the future, we will conduct in-depth clinical and experimental studies based on the results of our study.

Although we did not show the exact causal effect of prostate cancer with COVID-19, these results did not deny the role of prostate cancer during the susceptibility, hospitalization and mortality rates increased of COVID-19. In fact, more previous observational studies suggested the immunosuppressive effect of cancer and cancer-directed therapy increased the susceptibility of cancer patients to life-threatening complications from COVID-19.^[5,27]^ The development and progression of prostate cancer and COVID-19 are significantly influenced by common comorbidities such as hypertension, diabetes mellitus, obesity, old age, as well as molecular factors including androgen receptor signaling and TMPRSS2 expression. These factors collectively contributed to unfavorable outcomes.^[10]^ The presence of androgen, as well as the activation of androgen receptor signaling, a characteristic feature of prostate cancer, can exacerbate the severity of COVID-19 by enhancing the expression of TMPRSS2.^[28–31]^

TMPRSS2 can facilitate the activation of SARS-CoV-2, enabling viral entry into respiratory epithelial cells. Additionally, it functions as a cell surface protease that is regulated by androgens and normally overexpressed in prostate cancer tissues.^[32]^ Also, the androgen receptor serves as a crucial transcriptional regulator for TMPRSS2.^[33]^ Hence, the aggregation of risk and molecular factors between COVID-19 and prostate cancer renders individuals with prostate cancer a vulnerable population, predisposing them to an elevated susceptibility to severe COVID-19 infections, leading to increased rates of hospitalization and mortality.^[8]^ Furthermore, the protective effect of antiandrogen in reducing COVID-19 pathogenesis has been demonstrated by several studies, including the use of ADT.^[34]^ Therefore, the pursuit of TMPRSS2 as a rational therapeutic target to mitigate SARS-CoV2 infection was undertaken.^[35]^

In addition, an observational study has revealed that COVID-19 severity is positively associated with the occurrence of cytokine storms and increased levels of pro-inflammatory cytokines in peripheral blood.^[9]^ Relevant pro-inflammatory cytokines induced by COVID-19 can deteriorate prostate cancer.^[10]^ Nevertheless, in contrast to the reverse MR results, we found that there was inconsistent and contradictory to each other. Thus, the results we gained in this forward and reverse MR study drive us to make assumption that the interplay between COVID-19 and prostate cancer is attributed to inevitable confounding factors and initial physical circumstances. There is a complex relationship between prostate cancer and COVID-19, rather than a straightforward causal correlation. The connection between prostate cancer and COVID-19 may operate via other common pathways, rather than the diseases themselves. Moreover, it is hard to investigate the association employing the highest level of evidence in RCT study due to the enormous human power and financial resources. In the future, we are expected to combine Mendelian studies and meta-analyses to further study in different ancestries and multiple independent cohorts, thereby elucidating how prostate cancer affected the initiation and development of COVID-19.

For as we know, the current study represents the first MR study focusing on the causal effect between COVID-19 and prostate cancer. Several advantages of this MR study should be considered. Firstly, the sample sizes of both exposure and outcome were derived from the largest GWAS summary statistics, and compared to previous retrospective studies, this bidirectional MR could effectively reduce underlying bias including confounding factors and reverse causality. Secondly, it also assessed the causal link between prostate cancer and 3 diverse phenotypes of SARS-CoV2 infection, which supplied further insights into the causal links between prostate cancer and the severity of COVID-19. Thirdly, we further conducted a validation study through another independent cohort, which could ensure reliability of the results.

Inevitably, certain limitations exist in our research. First, although the results of the 5 analysis methods were robust, a small number of genetic instruments or underlying sample repetition between exposure and outcome may lead to bias. Second, to augment the statistical power, we should take advantage of the latest available data for COVID-19, which includes a much larger meta-analysis GWAS database. In this research, we only employ 3 phenotypes of COVID-19, and further research is needed. Third, as a result of the limited scope of MR, only the first assumption could be conventionally tested, while the validity of the remaining assumptions could not be guaranteed. Therefore, though we utilized multiple methods to eliminate evidence that is unlikely to hold up, there may still be residual bias. Fourth, although MR-egger and Cochran Q test indicated robustness between each SNP, the results of the leave-one-out method indicated there were still a few single SNPs potentially causing the results bias. Finally, GWAS could offer new insights into the genes implicated in exposure or outcome, but precise mechanisms studies are needed for a better understanding of the pathophysiology.

## 5. Conclusions

In conclusion, our research using bidirectional Mendelian Randomization revealed no causal association between the genetically predicted prostate cancer and COVID-19 phenotypes. Further research with various ancestries and large-scale datasets is encouraged to explore the causality and elucidate how prostate cancer affects the susceptibility, hospitalization, and severity of COVID-19 and how COVID-19 accelerates the progression of prostate cancer.

## Author contributions

**Conceptualization:** Renbing Pan.

**Data curation:** Renbing Pan.

**Funding acquisition:** Renbing Pan.

**Formal analysis:** Jingwen Liu.

**Investigation:** Jingwen Liu, Lijun Wan.

**Methodology:** Jingwen Liu, Lijun Wan, Jianyong Zhu.

**Software:** Jingwen Liu, Lijun Wan, Jianyong Zhu.

**Supervision:** Renbing Pan.

**Validation:** Jingwen Liu, Lijun Wan.

**Visualization:** Lijun Wan.

**Writing – original draft:** Jingwen Liu.

**Writing – review & editing:** Renbing Pan.

## Supplementary Material


