# Supplementary material for: Association between prostate cancer and susceptibility, hospitalization, and severity of COVID-19: Based on a Mendelian randomization study
Source: Medicine (Baltimore). 2024 Sep 6;103(36):e39430. doi: 10.1097/MD.0000000000039430 (PMC12431769; doi:10.1097/MD.0000000000039430)

**Figure S3:** Leave-one-out plot of the causal relationships between prostate cancer and COVID-19. Legend: (A) MR estimates for prostate cancer on COVID-19 susceptibility. (B) prostate cancer on COVID-19 hospitalization. (C) prostate cancer on COVID-19 severity. (D) MR estimates for COVID-19 susceptibility on prostate cancer. (E) COVID-19 hospitalization on prostate cancer. (F) COVID-19 severity on prostate cancer.


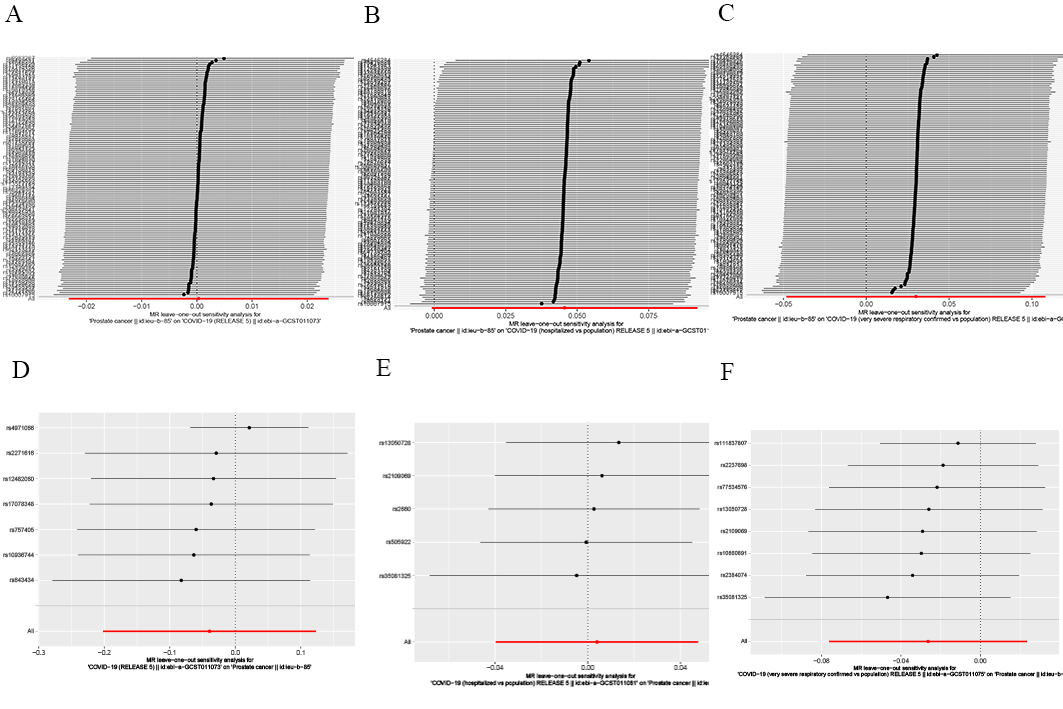

Supplement: Supplementary file 3 [file medi-103-e39430-s003.docx]
