# Supplementary material for: Association between prostate cancer and susceptibility, hospitalization, and severity of COVID-19: Based on a Mendelian randomization study
Source: Medicine (Baltimore). 2024 Sep 6;103(36):e39430. doi: 10.1097/MD.0000000000039430 (PMC12431769; doi:10.1097/MD.0000000000039430)
Supplement: Supplementary file 9 [file medi-103-e39430-s009.docx]

**Table S1.** Details of the genome-wide association studies and datasets used in this study.

| Characteristic | Source | Sample size | Ancestry | Access Link | PMID |
| --- | --- | --- | --- | --- | --- |
| Prostate cancer | PRACTICAL consortium | cases:79,148; controls:61,106 | European | http://gwas.mrcieu.ac.uk/datasets/ieu-b-85/ | 29892016 |
| COVID-19 susceptibility | COVID-19 Host Genetics Initiative | cases:38,984; controls:1,644,784 | European | http://gwas.mrcieu.ac.uk/datasets/ebi-a-GCST011073/ | 32404885 |
| COVID-19 hospitalization | COVID-19 Host Genetics Initiative | cases:9,986;  controls: 1,877,672 | European | http://gwas.mrcieu.ac.uk/datasets/ebi-a-GCST011081/ | 32404885 |
| COVID-19  severity | COVID-19 Host Genetics Initiative | cases:5,101; controls:1,383,241 | European | http://gwas.mrcieu.ac.uk/datasets/ebi-a-GCST011075/ | 32404885 |
| Prostate cancer | Biobank Japan (BBJ) | cases:5,408; controls:103,939 | East Asian | http://gwas.mrcieu.ac.uk/datasets/bbj-a-148/ | 32514122 |
