# Supplementary material for: Association between prostate cancer and susceptibility, hospitalization, and severity of COVID-19: Based on a Mendelian randomization study
Source: Medicine (Baltimore). 2024 Sep 6;103(36):e39430. doi: 10.1097/MD.0000000000039430 (PMC12431769; doi:10.1097/MD.0000000000039430)
Supplement: Supplementary file 13 [file medi-103-e39430-s013.docx]

| **Table S5.** MR estimate results of prostate cancer on COVID-19. | | | | | | | |  | |  | |  | |  | |  | |  | | |  | |  |  | |  |
| --- | --- | --- | --- | --- | --- | --- | --- | --- | --- | --- | --- | --- | --- | --- | --- | --- | --- | --- | --- | --- | --- | --- | --- | --- | --- | --- |
| **Outcome** | **Methods** | **nSNP** | **beta** | **SE** | ***P-* value** | **OR** | **or_lci95** | | **or_uci95** | | **Heterogeneity** | | | | | | | | | **Pleiotropy** | | | | |  |  |
|  |  |  |  |  |  |  |  |  |  |  | **MR-Egger** | | | | | | **IVW** | | | **Egger intercept** | | ***P-* value** | | |  |  |
|  |  |  |  |  |  |  |  |  |  |  | **Cochran’s *Q*** | | ***P*-value** | |  | | **Cochran’s *Q*** | | ***P*-value** |  |  |  |  |  |  |  |
| COVID-19  susceptibility | MR-Egger | 120 | -0.025 | 0.023 | 0.279 | 0.975 | 0.937 | | 1.020 | | 128.87 | | 0.161 | |  | | 130.74 | | 0.149 | 0.0029 | | 0.201 | | |  |  |
|  | Weighted median | 120 | -0.026 | 0.017 | 0.137 | 0.974 | 0.942 | | 1.008 | |  | |  | |  | |  | |  |  | |  | | |  |  |
|  | IVW | 120 | 0.0003 | 0.012 | 0.978 | 1.000 | 0.977 | | 1.024 | |  | |  | |  | |  | |  |  | |  | | |  |  |
|  | Simple mode | 120 | -0.007 | 0.039 | 0.986 | 0.999 | 0.925 | | 1.080 | |  | |  | |  | |  | |  |  | |  | | |  |  |
|  | Weighted mode | 120 | -0.030 | 0.020 | 0.142 | 0.970 | 0.932 | | 1.009 | |  | |  | |  | |  | |  |  | |  | | |  |  |
| COVID-19  hospitalization | MR-Egger | 120 | 0.023 | 0.047 | 0.619 | 1.024 | 0.934 | | 1.122 | | 124.25 | | 0.221 | |  | | 124.58 | | 0.234 | 4 0.0025 | | 0.581 | | |  |  |
|  | Weighted median | 120 | 0.064 | 0.035 | 0.071 | 1.066 | 0.995 | | 1.143 | |  | |  | |  | |  | |  |  | |  | | |  |  |
|  | IVW | 120 | 0.046 | 0.024 | 0.054 | 1.047 | 0.999 | | 1.096 | |  | |  | |  | |  | |  |  | |  | | |  |  |
|  | Simple mode | 120 | 0.109 | 0.068 | 0.113 | 1.115 | 0.976 | | 1.275 | |  | |  | |  | |  | |  |  | |  | | |  |  |
|  | Weighted mode | 120 | 0.071 | 0.044 | 0.110 | 1.074 | 0.985 | | 1.170 | |  | |  | |  | |  | |  |  | |  | | |  |  |
| COVID-19  severity | MR-Egger | 120 | -0.011 | 0.080 | 0.887 | 0.989 | 0.845 | | 1.157 | | 150.91 | | 0.012 | |  | | 151.38 | | 0.013 | 0.0046 | | 0.553 | | |  |  |
|  | Weighted median | 120 | 4.23e-05 | 0.058 | 0.999 | 1.000 | 0.893 | | 1.120 | |  | |  | |  | |  | |  |  | |  | | |  |  |
|  | IVW | 120 | 0.029 | 0.039 | 0.453 | 1.030 | 0.953 | | 1.114 | |  | |  | |  | |  | |  |  | |  | | |  |  |
|  | Simple mode | 120 | -0.146 | 0.141 | 0.304 | 0.864 | 0.656 | | 1.139 | |  | |  | |  | |  | |  |  | |  | | |  |  |
|  | Weighted mode | 120 | -0.042 | 0.084 | 0.618 | 0.959 | 0.814 | | 1.129 | |  | |  | |  | |  | |  |  | |  | | |  |  |

Abbreviations: SNP: single nucleotide polymorphism; SE: standard error of beta; IVW: Inverse variance weighted; OR: odd ratio.
