# Supplementary material for: Association between prostate cancer and susceptibility, hospitalization, and severity of COVID-19: Based on a Mendelian randomization study
Source: Medicine (Baltimore). 2024 Sep 6;103(36):e39430. doi: 10.1097/MD.0000000000039430 (PMC12431769; doi:10.1097/MD.0000000000039430)
Supplement: Supplementary file 14 [file medi-103-e39430-s014.docx]

| **Table S6.** MR estimate results of COVID-19 on prostate cancer. | | | | | | | |  | |  | |  | |  | |  | |  | | |  | |  |  | |  |
| --- | --- | --- | --- | --- | --- | --- | --- | --- | --- | --- | --- | --- | --- | --- | --- | --- | --- | --- | --- | --- | --- | --- | --- | --- | --- | --- |
| **Exposure** | **Methods** | **nSNP** | **beta** | **SE** | ***P-* value** | **OR** | **or_lci95** | | **or_uci95** | | **Heterogeneity** | | | | | | | | | **Pleiotropy** | | | | |  |  |
|  |  |  |  |  |  |  |  |  |  |  | **MR-Egger** | | | | | | **IVW** | | | **Egger intercept** | | ***P-* value** | | |  |  |
|  |  |  |  |  |  |  |  |  |  |  | **Cochran’s *Q*** | | ***P*-value** | |  | | **Cochran’s *Q*** | | ***P*-value** |  |  |  |  |  |  |  |
| COVID-19  susceptibility | MR-Egger | 7 | -0.070 | 0.310 | 0.829 | 0.932 | 0.507 | | 1.712 | | 21.60 | | 0.001 | |  | | 21.65 | | 0.001 | 0.0028 | | 0.920 | | |  |  |
|  | Weighted median | 7 | -0.027 | 0.056 | 0.634 | 0.974 | 0.873 | | 1.086 | |  | |  | |  | |  | |  |  | |  | | |  |  |
|  | IVW | 7 | -0.039 | 0.083 | 0.635 | 0.961 | 0.817 | | 1.131 | |  | |  | |  | |  | |  |  | |  | | |  |  |
|  | Simple mode | 7 | 0.010 | 0.077 | 0.899 | 1.010 | 0.868 | | 1.175 | |  | |  | |  | |  | |  |  | |  | | |  |  |
|  | Weighted mode | 7 | 0.022 | 0.069 | 0.761 | 1.022 | 0.892 | | 1.172 | |  | |  | |  | |  | |  |  | |  | | |  |  |
| COVID-19  hospitalization | MR-Egger | 5 | 0.005 | 0.043 | 0.914 | 1.005 | 0.923 | | 1.094 | | 1.172 | | 0.759 | |  | | 1.173 | | 0.882 | 4 -0.00024 | | 0.978 | | |  |  |
|  | Weighted median | 5 | 0.006 | 0.025 | 0.798 | 1.007 | 0.958 | | 1.058 | |  | |  | |  | |  | |  |  | |  | | |  |  |
|  | IVW | 5 | 0.004 | 0.022 | 0.859 | 1.004 | 0.961 | | 1.049 | |  | |  | |  | |  | |  |  | |  | | |  |  |
|  | Simple mode | 5 | 0.007 | 0.039 | 0.859 | 1.007 | 0.934 | | 1.087 | |  | |  | |  | |  | |  |  | |  | | |  |  |
|  | Weighted mode | 5 | 0.009 | 0.031 | 0.778 | 1.009 | 0.949 | | 1.074 | |  | |  | |  | |  | |  |  | |  | | |  |  |
| COVID-19  severity | MR-Egger | 8 | -0.011 | 0.068 | 0.881 | 0.989 | 0.866 | | 1.131 | | 21.44 | | 0.002 | |  | | 21.67 | | 0.003 | -0.0049 | | 0.809 | | |  |  |
|  | Weighted median | 8 | 3.88e-04 | 0.019 | 0.985 | 1.000 | 0.962 | | 1.040 | |  | |  | |  | |  | |  |  | |  | | |  |  |
|  | IVW | 8 | -0.026 | 0.025 | 0.300 | 0.974 | 0.927 | | 1.024 | |  | |  | |  | |  | |  |  | |  | | |  |  |
|  | Simple mode | 8 | 0.003 | 0.025 | 0.917 | 1.003 | 0.954 | | 1.053 | |  | |  | |  | |  | |  |  | |  | | |  |  |
|  | Weighted mode | 8 | 0.005 | 0.020 | 0.828 | 1.005 | 0.966 | | 1.045 | |  | |  | |  | |  | |  |  | |  | | |  |  |

Abbreviations: SNP: single nucleotide polymorphism; SE: standard error of beta; IVW: Inverse variance weighted; OR: odd ratio.
