# Supplementary material for: Association between prostate cancer and susceptibility, hospitalization, and severity of COVID-19: Based on a Mendelian randomization study
Source: Medicine (Baltimore). 2024 Sep 6;103(36):e39430. doi: 10.1097/MD.0000000000039430 (PMC12431769; doi:10.1097/MD.0000000000039430)
Supplement: Supplementary file 15 [file medi-103-e39430-s015.docx]

| **Table S7.** MR estimate results of prostate cancer on COVID-19. | | | | | | | |  | |  | |  | |  | |  | |  | | |  | |  |  | |  |
| --- | --- | --- | --- | --- | --- | --- | --- | --- | --- | --- | --- | --- | --- | --- | --- | --- | --- | --- | --- | --- | --- | --- | --- | --- | --- | --- |
| **Outcome** | **Methods** | **nSNP** | **beta** | **SE** | ***P-* value** | **OR** | **or_lci95** | | **or_uci95** | | **Heterogeneity** | | | | | | | | | **Pleiotropy** | | | | |  |  |
|  |  |  |  |  |  |  |  |  |  |  | **MR-Egger** | | | | | | **IVW** | | | **Egger intercept** | | ***P-* value** | | |  |  |
|  |  |  |  |  |  |  |  |  |  |  | **Cochran’s *Q*** | | ***P*-value** | |  | | **Cochran’s *Q*** | | ***P*-value** |  |  |  |  |  |  |  |
| COVID-19  susceptibility | MR-Egger | 22 | 0.023 | 0.026 | 0.393 | 1.023 | 0.972 | | 1.076 | | 18.45 | | 0.620 | |  | | 18.56 | | 0.672 | -0.0021 | | 0.746 | | |  |  |
|  | Weighted median | 22 | 0.005 | 0.015 | 0.758 | 1.005 | 0.975 | | 1.035 | |  | |  | |  | |  | |  |  | |  | | |  |  |
|  | IVW | 22 | 0.015 | 0.011 | 0.166 | 1.015 | 0.994 | | 1.037 | |  | |  | |  | |  | |  |  | |  | | |  |  |
|  | Simple mode | 22 | 0.007 | 0.021 | 0.738 | 1.007 | 0.967 | | 1.048 | |  | |  | |  | |  | |  |  | |  | | |  |  |
|  | Weighted mode | 22 | 0.002 | 0.018 | 0.904 | 1.002 | 0.968 | | 1.037 | |  | |  | |  | |  | |  |  | |  | | |  |  |
| COVID-19  hospitalization | MR-Egger | 22 | 0.043 | 0.021 | 0.044 | 1.044 | 1.001 | | 1.089 | | 13.23 | | 0.927 | |  | | 12.16 | | 0.935 | 4 -0.0129 | | 0.313 | | |  |  |
|  | Weighted median | 22 | 0.042 | 0.031 | 0.185 | 1.042 | 0.980 | | 1.109 | |  | |  | |  | |  | |  |  | |  | | |  |  |
|  | IVW | 22 | 0.092 | 0.052 | 0.091 | 1.096 | 0.990 | | 1.214 | |  | |  | |  | |  | |  |  | |  | | |  |  |
|  | Simple mode | 22 | 0.102 | 0.045 | 0.035 | 1.107 | 1.013 | | 1.211 | |  | |  | |  | |  | |  |  | |  | | |  |  |
|  | Weighted mode | 22 | 0.054 | 0.036 | 0.154 | 1.055 | 0.983 | | 1.134 | |  | |  | |  | |  | |  |  | |  | | |  |  |
| COVID-19  severity | MR-Egger | 22 | 0.144 | 0.080 | 0.086 | 1.155 | 0.987 | | 1.350 | | 18.43 | | 0.622 | |  | | 21.43 | | 0.494 | -0.0329 | | 0.098 | | |  |  |
|  | Weighted median | 22 | 0.043 | 0.050 | 0.388 | 1.044 | 0.947 | | 1.151 | |  | |  | |  | |  | |  |  | |  | | |  |  |
|  | IVW | 22 | 0.018 | 0.033 | 0.592 | 1.018 | 0.954 | | 1.085 | |  | |  | |  | |  | |  |  | |  | | |  |  |
|  | Simple mode | 22 | 0.064 | 0.083 | 0.444 | 1.067 | 0.907 | | 1.254 | |  | |  | |  | |  | |  |  | |  | | |  |  |
|  | Weighted mode | 22 | 0.064 | 0.057 | 0.271 | 1.067 | 0.954 | | 1.193 | |  | |  | |  | |  | |  |  | |  | | |  |  |

Abbreviations: SNP: single nucleotide polymorphism; SE: standard error of beta; IVW: Inverse variance weighted; OR: odd ratio.
