# Supplementary material for: Association between prostate cancer and susceptibility, hospitalization, and severity of COVID-19: Based on a Mendelian randomization study
Source: Medicine (Baltimore). 2024 Sep 6;103(36):e39430. doi: 10.1097/MD.0000000000039430 (PMC12431769; doi:10.1097/MD.0000000000039430)
Supplement: Supplementary file 16 [file medi-103-e39430-s016.docx]

| **Table S8.** MR estimate results of COVID-19 on prostate cancer. | | | | | | | |  | |  | |  | |  | |  | |  | | |  | |  |  | |  |
| --- | --- | --- | --- | --- | --- | --- | --- | --- | --- | --- | --- | --- | --- | --- | --- | --- | --- | --- | --- | --- | --- | --- | --- | --- | --- | --- |
| **Exposure** | **Methods** | **nSNP** | **beta** | **SE** | ***P-* value** | **OR** | **or_lci95** | | **or_uci95** | | **Heterogeneity** | | | | | | | | | **Pleiotropy** | | | | |  |  |
|  |  |  |  |  |  |  |  |  |  |  | **MR-Egger** | | | | | | **IVW** | | | **Egger intercept** | | ***P-* value** | | |  |  |
|  |  |  |  |  |  |  |  |  |  |  | **Cochran’s *Q*** | | ***P*-value** | |  | | **Cochran’s *Q*** | | ***P*-value** |  |  |  |  |  |  |  |
| COVID-19  susceptibility | MR-Egger | 6 | 0.505 | 0.379 | 0.254 | 1.657 | 0.787 | | 3.487 | | 1.199 | | 0.878 | |  | | 1.629 | | 0.898 | -0.0236 | | 0.548 | | |  |  |
|  | Weighted median | 6 | 0.296 | 0.149 | 0.048 | 1.345 | 1.003 | | 1.803 | |  | |  | |  | |  | |  |  | |  | | |  |  |
|  | IVW | 6 | 0.292 | 0.161 | 0.129 | 1.339 | 0.976 | | 1.838 | |  | |  | |  | |  | |  |  | |  | | |  |  |
|  | Simple mode | 6 | 0.273 | 0.198 | 0.226 | 1.314 | 0.891 | | 1.938 | |  | |  | |  | |  | |  |  | |  | | |  |  |
|  | Weighted mode | 6 | 0.269 | 0.120 | 0.025 | 1.308 | 1.034 | | 1.656 | |  | |  | |  | |  | |  |  | |  | | |  |  |
| COVID-19  hospitalization | MR-Egger | 4 | -0.950 | 0.532 | 0.216 | 0.387 | 0.136 | | 1.097 | | 2.644 | | 0.267 | |  | | 6.372 | | 0.095 | 4 0.1241 | | 0.235 | | |  |  |
|  | Weighted median | 4 | -0.108 | 0.114 | 0.344 | 0.898 | 0.719 | | 1.122 | |  | |  | |  | |  | |  |  | |  | | |  |  |
|  | IVW | 4 | -0.072 | 0.124 | 0.559 | 0.930 | 0.729 | | 1.186 | |  | |  | |  | |  | |  |  | |  | | |  |  |
|  | Simple mode | 4 | 0.123 | 0.234 | 0.636 | 1.131 | 0.715 | | 1.788 | |  | |  | |  | |  | |  |  | |  | | |  |  |
|  | Weighted mode | 4 | -0.229 | 0.117 | 0.145 | 0.795 | 0.632 | | 1.000 | |  | |  | |  | |  | |  |  | |  | | |  |  |
| COVID-19  severity | MR-Egger | 7 | 0.043 | 0.264 | 0.875 | 1.044 | 0.623 | | 1.752 | | 7.231 | | 0.204 | |  | | 7.761 | | 0.256 | -0.0386 | | 0.571 | | |  |  |
|  | Weighted median | 7 | -0.169 | 0.072 | 0.018 | 0.844 | 0.734 | | 0.972 | |  | |  | |  | |  | |  |  | |  | | |  |  |
|  | IVW | 7 | -0.112 | 0.059 | 0.062 | 0.894 | 0.795 | | 1.006 | |  | |  | |  | |  | |  |  | |  | | |  |  |
|  | Simple mode | 7 | -0.251 | 0.130 | 0.102 | 0.778 | 0.603 | | 1.004 | |  | |  | |  | |  | |  |  | |  | | |  |  |
|  | Weighted mode | 7 | -0.216 | 0.099 | 0.073 | 0.806 | 0.663 | | 0.979 | |  | |  | |  | |  | |  |  | |  | | |  |  |

Abbreviations: SNP: single nucleotide polymorphism; SE: standard error of beta; IVW: Inverse variance weighted; OR: odd ratio.
